# Reduced maximal range of ocular movements and its response to acute levodopa challenge in Parkinson's disease

**DOI:** 10.3389/fnagi.2024.1368539

**Published:** 2024-03-20

**Authors:** Juan Li, Yuewen Li, Xianzhou Chu, Mengxue Jiang, Tieyu Wu, Xianwen Chen

**Affiliations:** ^1^Department of Neurology, The First Affiliated Hospital of Anhui Medical University, Hefei, China; ^2^Department of Neurology, The Second Affiliated Hospital of Anhui Medical University, Hefei, China

**Keywords:** Parkinson's disease, oculomotor range, progressive supranuclear palsy, multiple system atrophy, differential diagnosis

## Abstract

**Introduction:**

Although restriction of vertical ocular range of motion is known to be the hallmark of progressive supranuclear palsy (PSP), the maximal amplitude of ocular movement has not been quantitatively assessed despite of accumulating evidences of oculomotor dysfunction in Parkinson's disease (PD). Here, we evaluated the maximal oculomotor range and its response to levodopa in PD, and compare findings to atypical parkinsonism.

**Methods:**

We recruited 159 healthy controls (HC) as well as 154 PD, 30 PSP, and 16 multiple system atrophy (MSA) patients. Oculomotor range was assessed using a kinetic perimeter-adapted device for the vertical and horizontal axes (four positions). Parameters were reassessed after levodopa challenge and compared among PD, PSP, and MSA patients.

**Results:**

Maximum oculomotor range in PD patients was reduced as compared to HC. Levodopa improved oculomotor range in all directions; corrective effects of upward range positively correlated with improvements in Unified Parkinson's Disease Rating Scale III and bradykinesia sub-scores among PD patients. Although oculomotor range was markedly restricted among PSP and MSA patients, the beneficial effects of levodopa was less pronounced. Reduced oculomotor range of motion was more significant among PSP as compared to PD or MSA patients; MSA patients did not significantly differ from PD patients. The range of upward gaze was optimally sensitive for differentiating among PD, PSP, and MSA patients.

**Conclusion:**

Maximum oculomotor range was reduced among PD patients significantly improved by levodopa treatment. Variations in, as well as the positively effects of levodopa on, the range of upward gaze assist diagnostic differentiation among PD, PSP, and MSA patients.

## 1 Introduction

The diagnosis of Parkinson's disease (PD)relies on clinical assessments of cardinal motor symptoms such as bradykinesia, rigidity, tremor, and postural instability. Ocular movements in this patient population, however, are often overlooked due to their subtle nature and relatively low impact on overall disease burden. Interestingly, the Movement Disorder Society-sponsored Revision of the Unified Parkinson's Disease Rating Scale (MDS-UPDRS) does not include a specific criterion for evaluating ocular movements. Previous studies on eye movements of PD patients have primarily focused on saccades, smooth pursuit and abnormal movement patterns, with saccades being the most extensively investigated. Techniques such as video-based eye tracking, electro-oculography and videonystagmography have been utilized to study hypometric saccades, reduced saccade velocity and prolonged saccade latency in people with PD (Zhang et al., 2018; Zhou et al., 2022). These studies about saccades, however, only implied the magnitude of change overtime, limited attention are looking simply at the magnitude or gain of ocular movements at a fixed time. In clinical practice, physicians typically consider evaluation of eye movements in cases of atypical presentations, particularly when there is suspicion of progressive supranuclear palsy (PSP).

Atypical parkinsonian syndromes, such as multiple system atrophy (MSA) and PSP, are the next most prevalent forms of neurodegenerative parkinsonism after classical PD. Despite obvious symptomatic differences, it remains challenging to distinguish classical from atypical PD, especially early in the disease course. Presence of ocular signs and eye movement deficits thus provide valuable guidance in establishing accurate diagnosis (Anderson and MacAskill, 2013). For instance, abnormal fixation and square-wave jerks are frequently observed in MSA patients (Rascol et al., 1991, 1995), with the presence of cerebellar-type oculomotor disturbances, such as gaze-evoked nystagmus, downbeat positioning nystagmus, optokinetic nystagmus, and vestibulo-ocular reflex suppression strongly supporting a diagnosis of MSA (Anderson et al., 2010). Vertical gaze dysfunction is commonly seen in clinic and considered to be highly suggestive of PSP (Höglinger et al., 2017). However, the extent to which ocular range of motion is limited in PSP patients has not been clarified, especially in relation classical and atypical PD settings. Although accumulating data have underscored the presence of oculomotor deficits in PD patients, whether maximal oculomotor range is altered among such patients remains unknown. Importantly, differences in maximum oculomotor range between classical and atypical PD syndromes, as well as the influence of levodopa on eye movement has not been explored in the context of differentiating among PD, PSP and MSA diagnoses.

To meet clinical requirements, it is essential to develop an accurate, reliable, and user-friendly method for evaluating the maximum range of ocular motion. Regrettably, there is currently no standardized approach for assessing this parameter in either research or clinical settings. Previous studies used the light reflex or perimeters to measure the range of eye movement (Chamberlain, 1971; Gerling et al., 1997; Haggerty et al., 2005). Findings obtained via these methods, however, tend to be affected by the examiner's experience and are thus difficult to standardize. Lee et al. (2019) described a method to quantify the angle of eye movement by computer-assisted analysis of photographs of eyes in different gaze positions (Lim et al., 2014). Even so, the use of this method requires specialized photographic equipment and software, which makes its widespread implementation highly impractical. The scleral search coil method and video-oculography based on pupil tracking and corneal reflection have been considered as the gold standard for accurate eye movement measurement (van der Geest and Frens, 2002). These instruments, however, are not specifically designed for measuring the maximum range of ocular movement accurately and reliably. For example, the use of search coils embedded within contact lenses has primarily focused on the detection and characterization of nystagmus, such patients should ideally avoid extreme gazes due to interference of eyelids with delicate wire connections and the risk of contact lens dislodging or artifact introduction into signal data (Frens and van der Geest, 2002; Smeets and Hooge, 2003). Furthermore, such devices are not widely available and remain laborious to utilize. There is thus a need to establish a simple and reliable method for measuring maximum ocular motion range that is applicable in routine clinical examination. Here, we describe a method for rapid evaluation of eye movement modified from perimeter testing, using indication bars and a marked scale to accurately measure maximum tracking range from the point of origin to extreme gaze positions.

The aims of this study were to: (1) quantify the maximum oculomotor range in PD patients via a simple and rapid measuring method and analyze the differences among HC, PD, PSP, and MSA patients (2) investigate the effect of acute levodopa challenge on the range of ocular motion and assess the diagnostic value of such data for different parkinsonian disorders.

## 2 Methods

### 2.1 Participants

A total of 359 participants were enrolled in this study and included 154 PD patients, 30 PSP patients, 16 MSA patients, and 159 healthy controls (HC). The PSP cohort consisted of 17 patients suffering the PSP-parkinsonism (PSP-P) subtype and 13 suffering the PSP Richardson's syndrome (PSP-RS) subtype. All MSA patients were of the Parkinson type (MSA-P). Diagnoses were established by an experienced neurologist based on established clinical standards (Hughes et al., 1992; Gilman et al., 2008; Höglinger et al., 2017). Participants with any known ocular disease, apparent deficits in vision or other neurological and psychiatric conditions were excluded from analyses. All participants confirmed that they were able to clearly see visual targets with a corrected Snellen visual acuity of 20/60 or better in the worse eye. The experimental procedure was approved by the ethical committee of the First Affiliated Hospital of Anhui Medical University; all participants provided written informed consent prior to commencement of experimentation. This study was registered in the Chinese Clinical Trial Register Center (ChiCTR2300070333).

### 2.2 Clinical assessments

Demographic data including age, gender and disease course were collected. All patients refrained from taking any antiparkinsonian medications overnight prior to baseline assessment. Hoehn and Yahr (H-Y) stage, levodopa equivalent daily dose (LEDD), Part II and Part III MDS-UPDRS scores (MDS-UPDRS II and MDS-UPDRS III) were recorded for each patient. Sub-scores of motor functions derived from relevant MDS-UPDRS III items were calculated respectively: rigidity (item 3); bradykinesia (items 4–8, 14), gait and posture (items 9–13), tremor (items 15–18).

### 2.3 Instrumentation used for oculomotor range evaluation

**Figure 1:**
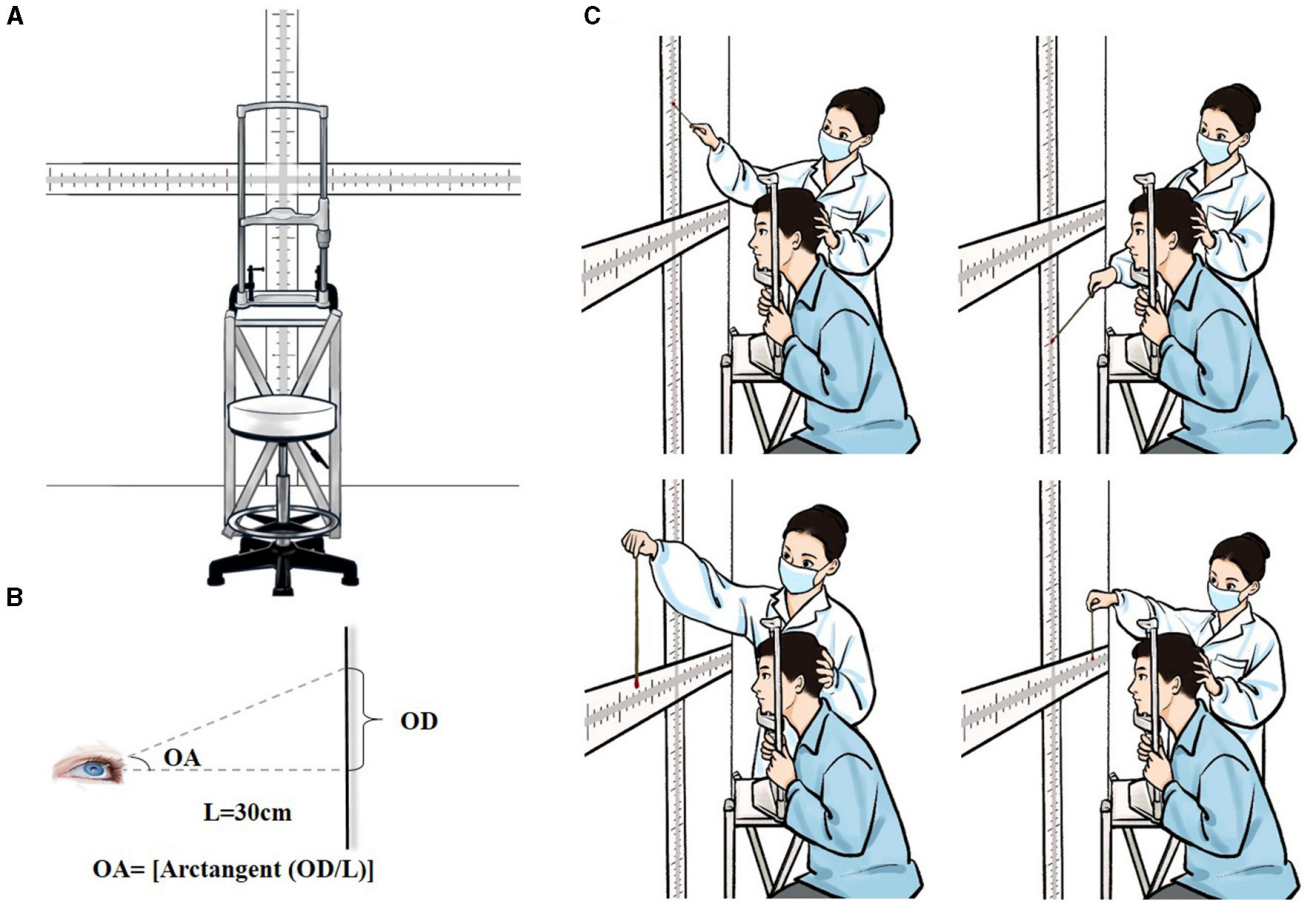
An illustration of the test setup and scene. **(A)** Specially-designed device for measuring maximum oculomotor range. **(B)** Detection of the angle of upward gaze. L, the distance between the zero point and the midpoint between two eyes; OD, the oculomotor distance, which was the distance from the zero point to the position indicated by the stick at the extreme of the upward gaze on the marked scale; OA, the oculomotor angle, OA = [arctangent (OD/L)]. **(C)** Diagram of the test scene. The entire procedure comprised measurements of maximum ocular movements in all four directions (i.e., upward, downward, leftward and rightward).

The instrument used for quantification of oculomotor range consisted of cruciate-shaped scale with vertical and horizontal components, a stand equipped with a head holder, and a height-adjustable chair (Figure 1A). A marked scale was fixed on the wall facing the stand. The primary visual target point (zero point) was set at the intersection of horizontal and vertical scale sections with a value of “0.” The scale extended outward from the point of zero along four axes (i.e., upward, downward, leftward and rightward), with graded values that ranged from 0 to 100 cm at a precision of 1 mm. A stand with a height-adjustable head holder was placed between the wall and the test subject. Chair and head holder was adjusted to align the scale's zero point with the middle point of the subject's eyes on a level plane. The distance between the zero point and the middle point of the subject's eyes was maintained at 30 cm (Length, L = 30 cm); the axis passing through these two points was adjusted to be perpendicular to the wall (Figure 1A).

### 2.4 Measurement procedure

Participants were introduced to test procedures before formal testing commenced. The test was performed in a well-lit room. Subjects were seated on a height-adjustable chair, facing the marked scale mounted on the wall. Subjects were asked to sit with their legs apart, back straight and head positioned on the chin rest with the middle point between their two eyes aligned with the scale's zero point on a level plane. Head position was examined to ensure stability during testing. The examiner held a marked indication stick (diameter 0.5 cm) with colored ends (two distinct colors at each end), moving the stick from the outermost end of the scale toward the zero point and inquiring whether the subjects could identify the color at the stick's end. We selected an appropriately-sized ball with a diameter of 1 cm based on prior experience, avoiding the situation that the ball was too small to affect patients' visibility or too large to make accurate differential measurements. Participants were asked to fixate their gaze on the scale's zero point and subsequently move their eyes upward, downward, leftward and rightward as much as possible and try to visually trace the colored end of the stick. To avoid misreporting of data, indication sticks with different colors at each end were switched randomly without informing the subject during testing. The balls were selected from 5 colors: red, yellow, blue, green, and black, which were respectively represented by the numbers 1 to 5. A two-digit number is randomly generated by the computer using the digits 1, 2, 3, 4, and 5 without repetition. Two colored balls were chosen in each test according to the randomized number. The position of the stick's end relative to the scale was recorded as soon as the subject reported the color of the stick's end correctly on binocular gaze. This marked point on the scale was then recorded as the finial oculomotor distance (OD). To ensure data homogeneity and reproducibility for inter-laboratory comparisons, OD data was converted into those for oculomotor angle (OA). For convenience, we considered the distance from the point midway between the two eyes (i.e., the nose bridge) to the zero point of the marked scale (instead of the distance from each eye to the zero-point) as the “L” for calculating binocular gaze OA. As detailed in Figure 1B, OA was calculated as follows: OA = [arctangent (OD/L)]. All tests were performed by the same examiner who remained blind to subject group and medical conditions. Testing producers were repeated twice for all four gaze directions with the average value recorded as the final measurements (Figure 1C). Prior to the experiment, test-retest reliability was assessed in a cohort of 15 healthy individuals with an interval of 1 week by the same evaluator. The test-retest reliability was satisfactory since the correlation coefficients were above 0.50 for maximal oculomotor angles in all directions. Fifteen HC were each assessed by two independent observers and the *Kendall* Correlation Coefficient (*Kendall's W*) was applied to analyze inter-rater reliability. All oculomotor range measurements were statistically significant (*P* < 0.05), confirming that the results had high validity.

### 2.5 Acute levodopa challenge

Patients underwent an acute levodopa challenge after baseline assessment. A single tablet of levodopa/benserazide (200 mg levodopa/50 mg benserazide; Shanghai Roche Pharmaceuticals Ltd, Shanghai, China) was orally administered to patients with 100 ml of water. To achieve the maximum plasma concentration within the limited time frame of the clinic visit, we encouraged patients to thoroughly chew the tablet. The MDS-UPDRS III scores and maximal range of ocular movement were reassessed 1 h after drug administration.

### 2.6 Statistical analysis

Statistical analyses were performed using SPSS software version 26.0 (IBM Inc, Armonk, USA). Prior to statistical testing, data were assessed for normality and homogeneity of variance using either the *Kolmogorov-Smirnov* test or *Shapiro-Wilk*, based on the sample size. Clinical assessments including disease course, H-Y stage, LEDD, MDS-UPDRS II, MDS-UPDRS III, and relevant sub-scores were also compared among PD, PSP, and MSA patient groups. Continuous variables that followed a normal distribution were analyzed using the ANOVA test. Non-normally distributed variables were analyzed using the *Kruskal-Wallis* test. *Post-hoc* comparisons were performed for the cross-sectional oculomotor range between HC and the three patient groups using *Bonferroni* correction (*P* < 0.05/4 i.e., < 0.0125). Categorical data were evaluated using the *chi-squared* test. Changes in scale and oculomotor range after levodopa challenge within each group were assessed using a paired *t-*test or Wilcoxon signed-rank test. The threshold for significance was set at *P* < 0.05.

## 3 Results

### 3.1 Participants characteristics

Basic demographic and clinical data are presented in Table 1. For people with PSP, mean age, LEDD, MDS-UPDRS II, and MDS-UPDRS III values were significantly higher as compared to those of PD patients. Sub-scores related to bradykinesia, posture and gait were significantly higher in PSP patients, while tremor scores were much lower. Differences in rigidity scores between PD and PSP patients were not significant. Compared to PD patients, MSA patients had a slightly higher proportion of females than males but this did not reach statistical significance. MDS-UPDRS II scores were higher in MSA as compared to PD patients. Despite similar MDS-UPDRS III scores, MSA patients exhibited higher scores for posture and gait but lower scores for tremor as compared to PD patients.

**Table 1:** Baseline demographics.

**Measurements**	**HC (*n =* 159)**	**PS (*****n** =* **200)**			***Z/F/*χ**	***P*-value**
		**PD (*****n** =* **154)**	**PSP (*****n** =* **30)**	**MSA (*****n** =* **16)**		
Age (years)	61.73 ± 9.15	62.14 ± 9.39	69.37 ± 5.91*^∧^*	62.19 ± 8.49	18.293	< 0.001
Gender (M/F)	88/71	92/62	18/12	6/10	3.234	0.357
Disease duration (months)	-	40.21 ± 29.26	48.80 ± 34.33	32.13 ± 18.09	3.643	0.162
H-Y stage (I/II/III/IV/V)	-	34/78/40/2/0	0/6/20/4/0	0/4/8/4/0	51.668	< 0.001
LEDD (mg/day)	-	233.77 ± 264.89	384.33 ± 272.10^*^	375.00 ± 318.86	9.442	0.009
MDS-UPDRS II	-	10.37 ± 5.48	19.80 ± 7.01^*^	20.25 ± 7.23^#^	57.898	< 0.001
MDS-UPDRS III	-	33.93 ± 15.30	44.80 ± 15.54^*^	41.88 ± 15.56	7.483	0.001
Rigidity	-	6.73 ± 3.82	7.40 ± 4.58	7.06 ± 4.27	0.617	0.734
Bradykinesia	-	16.56 ± 8.09	22.40 ± 9.16^*^	21.63 ± 8.74	14.493	0.001
Posture and gait	-	3.64 ± 3.20	10.83 ± 4.42^*^	8.94 ± 4.54^#^	66.269	< 0.001
Tremor	-	6.23 ± 4.57	2.37 ± 3.34^*^	1.56 ± 2.42^#^	34.326	< 0.001

HC, healthy controls; PS, people with parkinsonism; PD, Parkinson's disease; PSP, progressive supranuclear palsy; MSA, multiple system atrophy; H-Y stage, Hoehn and Yahr stage; LEDD, levodopa equivalent daily dose; MDS-UPDRS II, Part II of the Movement Disorder Society-sponsored Revision of the Unified Parkinson's Disease Rating Scale; MDS-UPDRS III, Part III of the Movement Disorder Society-sponsored Revision of the Unified Parkinson's Disease Rating Scale. Comparisons among groups were *Bonferroni* adjusted. ^∧^significant difference between PSP and all the other groups with all *P* values < 0.0125 (*P* < 0.05/4 i.e., < 0.0125); ^*^significant difference between PD and PSP with *P* values < 0.017 (*P* < 0.05/3 i.e., < 0.017); ^#^significant difference between PD and MSA with *P* values < 0.017 (*P* < 0.05/3 i.e., < 0.017).

### 3.2 Range of ocular movements

**Figure 2:**
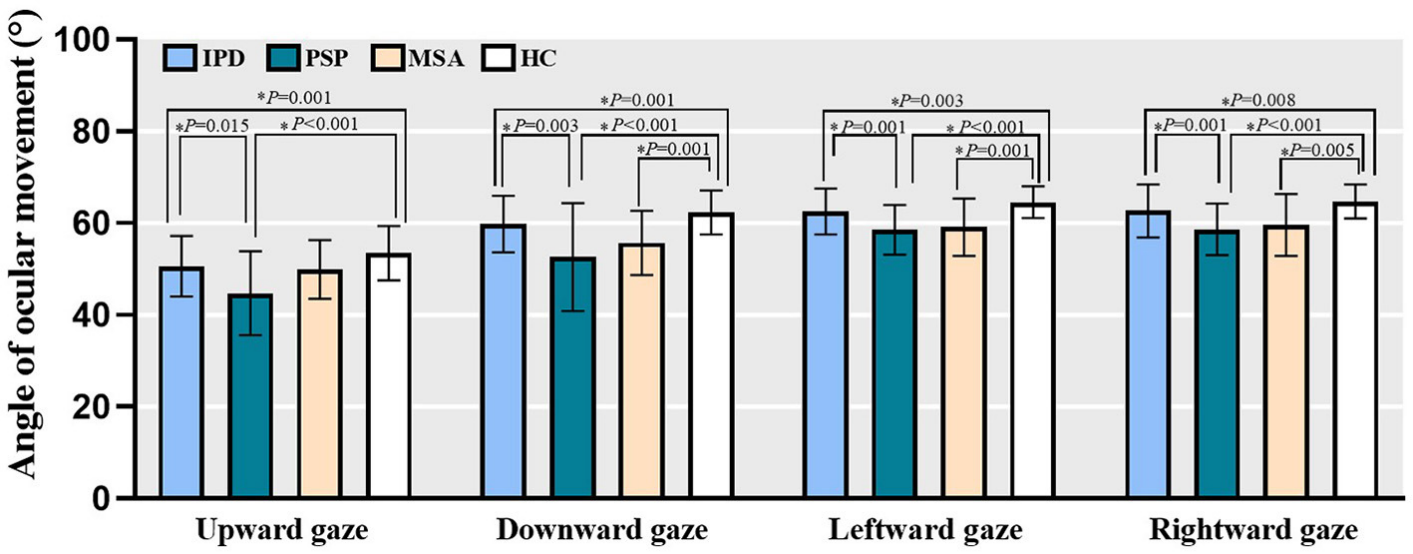
The maximum oculomotor angle of all four directions in PD, PSP, MSA patients as well as HC subjects. Bars represent mean value and error bars represent standard error. PD, people with Parkinson's disease; PSP, people with progressive supranuclear palsy; MSA, people with multiple system atrophy; HC, healthy controls. *P < 0.05.

Among HC subjects, the maximum angles of ocular movement for each position were 53.46 ± 5.95° for upward gaze, 62.33 ± 4.77° for downward gaze, 64.56 ± 3.45° for leftward gaze, and 64.75 ± 3.73° for rightward gaze. Compared to HC data, both PD and PSP subjects exhibited reduced maximum oculomotor angles for all four directions (People with PD: upward gaze, *Z* = −3.752, *P* = 0.001 All *P* < 0.05; downward gaze, *Z* = –3.703, *P* = 0.001; leftward gaze, *Z* = –3.441, *P* = 0.003; rightward gaze, *Z* = –3.220, *P* = 0.008; People with PSP: upward gaze, *Z* = –5.174, *P* < 0.001; downward gaze, *Z* = –5.641, *P* < 0.001; leftward gaze, *Z* = –5.725, *P* < 0.001; rightward gaze, *Z* = –5.738, *P* < 0.001); MSA patients similarly exhibited decreased downward and horizontal gaze angles (downward gaze, *Z* = –3.795, *P* = 0.001; leftward gaze, *Z* = –3.744, *P* = 0.001; rightward gaze, *Z* = –3.361, *P* = 0.005). People with PSP exhibited more severe maximum vertical and horizontal deficits relative to PD patients (upward gaze, *Z* = 3.032, *P* = 0.015; downward gaze, *Z* = 3.529, *P* = 0.003; leftward gaze, *Z* = 3.761, *P* = 0.001; rightward gaze, *Z* = 3.898, *P* = 0.001). No significant difference between PD and MSA patients was found (Figure 2).

### 3.3 Improvements after levodopa challenge

**Figure 3:**
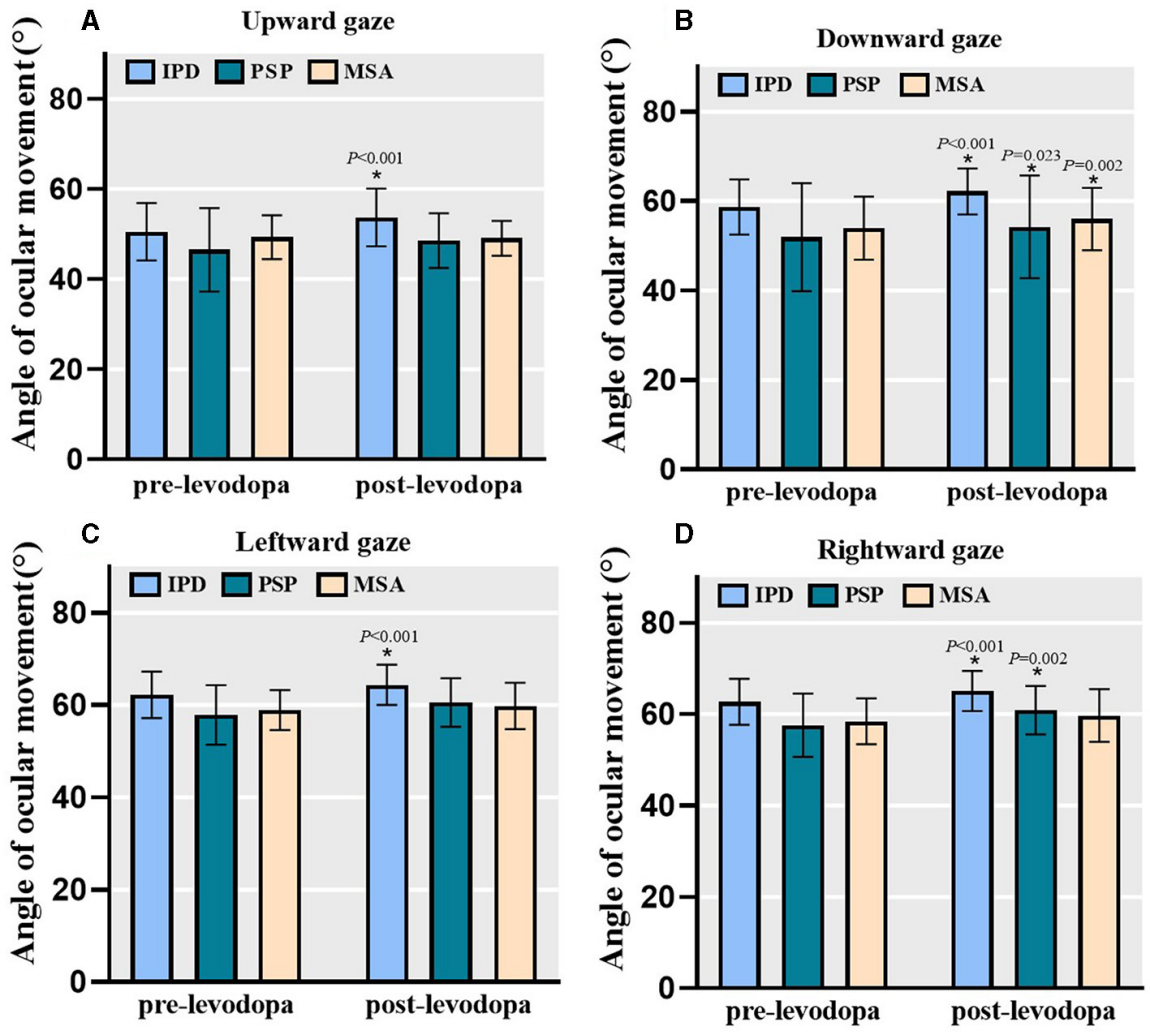
The angle of ocular movements at baseline and after levodopa challenge. Levodopa challenge significantly improved the maximum oculomotor angle of both horizontal and vertical gaze in people with PD (*n* = 86). For PSP group (*n* = 15), there were significant improvements for downward and rightward gaze but not for upward and leftward gaze. For MSA group (*n* = 10), only downward gaze significantly improved. Bars represent mean value and error bars represent standard error. PD, Parkinson's disease; PSP, progressive supranuclear palsy; MSA, multiple system atrophy. **(A)** Angle of ocular movement in upward gaze. **(B)** Angle of ocular movement in downward gaze. **(C)** Angle of ocular movement in leftward gaze. **(D)** Angle of ocular movement in rightward gaze. *P < 0.05.

In total, 111 [including 86 PD, 15 PSP (9 PSP-P, 6 PSP-RS), and 10 MSA] patients successfully underwent acute levodopa challenge and subsequent assessments. The primary factors that contribute to patients not undergoing the levodopa challenge included outpatient visitation time constrations, compromised physical condition, and adverse drug reactions. Pre- and post-levodopa challenge within-group effects were analyzed and changes in MDS-UPDRS III scores and relevant sub-scores determined (Table 2). Levodopa challenge demonstrated significant effects on overall motor functions and each subtype motor symptoms across all three groups with the exception of tremor in individuals with MSA. Levodopa challenge was found to significantly improve the maximum angle of ocular motion on both horizontal and vertical gaze among people with PD (Figure 3). Among PSP patients, significant improvements in downward and rightward maximal gaze was noted, although not in upward and leftward gaze. Among MSA patients, only downward gaze improved significantly. We compared the post-challenge outcomes in people with PD with those of HC, it is inspiring to find that there were no statistical differences in both the upward gaze, downward gaze and rightward gaze in these two groups (upward gaze, *F* = 0.576, *P* = 0.449; downward gaze, *Z* = –0.083, *P* = 0.934; rightward gaze, *Z* = –1.217, *P* = 0.223).

**Table 2:** Motor function assessment in enrolled patients (*n* = 111).

**Group**	**Motor symptoms**	**Pre-levodopa**	**Post-levodopa**	***t*/*Z***	***P*-value**
PD (*n =* 86)	MDS-UPDRS III	34.50 ± 14.16	20.81 ± 10.25	−8.057	< 0.001^*^
	Rigidity	6.57 ± 3.79	3.87 ± 2.98	−7.766	< 0.001^*^
	Bradykinesia	17.00 ± 8.00	10.01 ± 5.81	−7.790	< 0.001^*^
	Posture and gait	2.80 ± 2.45	2.05 ± 1.91	−5.467	< 0.001^*^
	Tremor	6.28 ± 4.45	3.16 ± 3.09	−7.191	< 0.001^*^
PSP (*n =* 15)	MDS-UPDRS III	44.60 ± 16.43	33.80 ± 14.67	7.358	< 0.001^*^
	Rigidity	8.13 ± 4.98	7.00 ± 4.60	2.828	0.013^*^
	Bradykinesia	22.33 ± 9.28	16.53 ± 8.07	7.360	< 0.001^*^
	Posture and gait	8.20 ± 3.65	6.33 ± 3.64	−2.988	0.003^*^
	Tremor	3.00 ± 3.85	1.47 ± 2.00	−2.214	0.027^*^
MSA (*n =* 10)	MDS-UPDRS III	47.40 ± 10.31	39.20 ± 8.54^*^	5.904	< 0.001^*^
	Rigidity	9.10 ± 2.96	6.80 ± 3.99^*^	3.851	0.004^*^
	Bradykinesia	25.00 ± 4.97	20.80 ± 3.71^*^	4.583	0.001^*^
	Posture and gait	7.80 ± 3.94	6.80 ± 3.46^*^	2.535	0.032^*^
	Tremor	2.10 ± 2.73	1.40 ± 2.32	−1.841	0.066

PD, Parkinson's disease; PSP, progressive supranuclear palsy; MSA, multiple system atrophy; MDS-UPDRS III, Part III of the Movement Disorder Society-sponsored Revision of the Unified Parkinson's Disease Rating Scale; ^*^significant difference between pre-challenge and post-challenge level with *P* value < 0.05.

### 3.4 Correlation analysis

**Figure 4:**
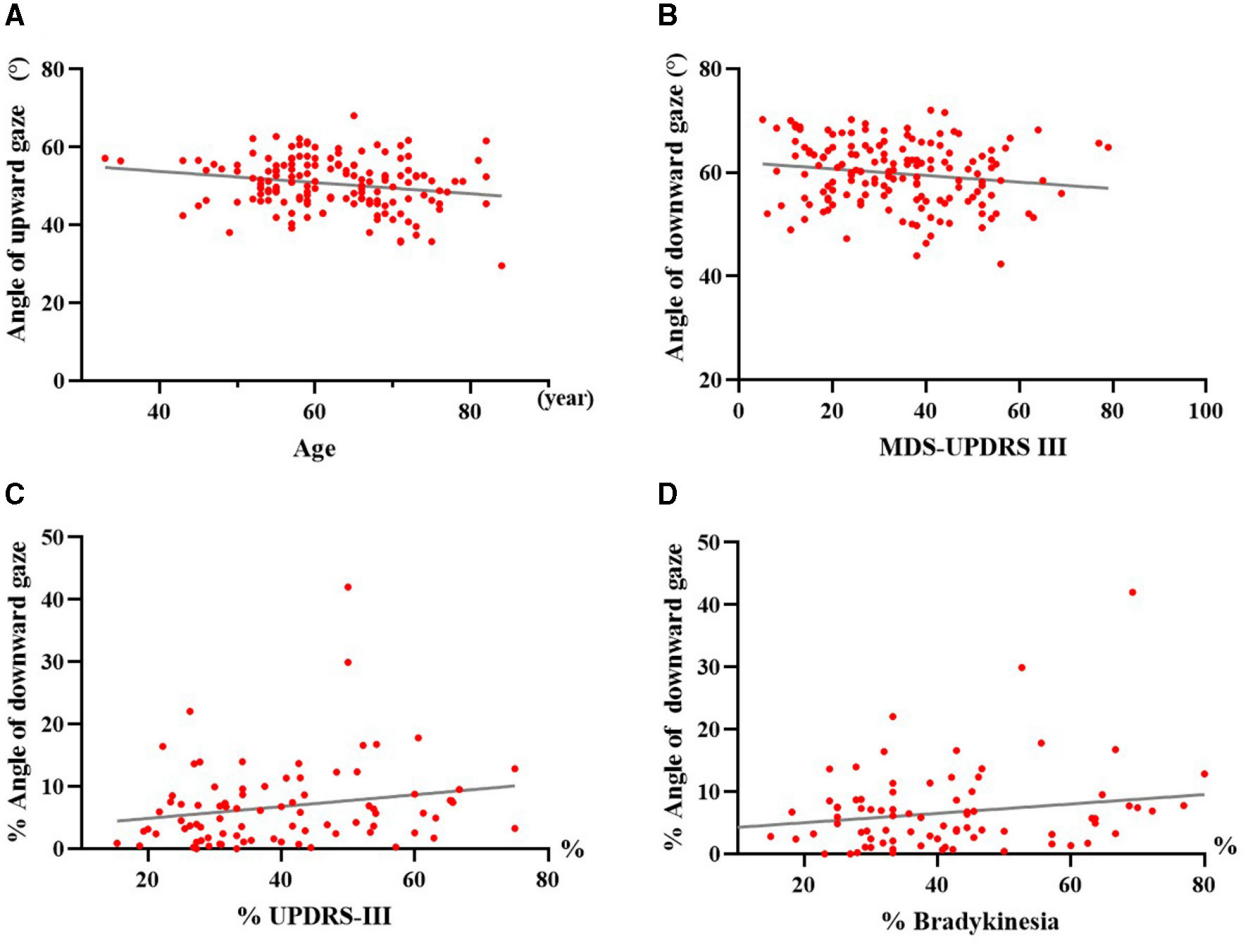
Correlation analyses of maximal oculomotor angle with age, motor symptoms and acute levodopa challenge in PD patients. **(A)** Correlation between maximum upward angle and age in PD. **(B)** Correlation between maximum downward angle and MDS-UPDRS III scores in PD before medication. **(C)** Correlation between the improvement ratio of maximum downward angle and MDS-UPDRS III scores in PD after acute levodopa challenge. **(D)** Correlation between the improvement ratio of maximum downward angle and bradykinesia sub-scores in PD after acute levodopa challenge. PD, people with Parkinson's disease; MDS-UPDRS III, Part III of the Movement Disorder Society-sponsored Revision of the Unified Parkinson's Disease Rating Scale; %, improvement ratio = (post-levodopa challenge data – pre-levodopa challenge data)/pre-levodopa challenge data × 100%. Each dot represents one patient.

At baseline, both HC and PD patients exhibited significant negative correlations between age and ocular movements for all directions (*P* < 0.05; PD: upward gaze, *r* = −0.205, *P* = 0.011; downward gaze, *r* = −0.247, *P* = 0.002; leftward gaze, *r* = –0.203, *P* = 0.011; rightward gaze, *r* = –0.320, *P* < 0.001; HC: upward gaze, *r* = –0.298, *P* < 0.001; downward gaze, *r* = –0.252, *P* = 0.001; leftward gaze, *r* = –0.200, *P* = 0.011; rightward gaze, *r* = −0.250, *P* < 0.001). A representative example revealed the maximal range of upward gaze to have negatively associated with age in PD patients (Figure 4A). The MDS-UPDRS III scores at baseline in people with PD were negatively correlated with the maximum oculomotor angle in downward gaze (Figure 4B). Among PD patients, there were significant positive correlations (*P* < 0.05) between the ratio of improvement in maximal downward range and MDS-UPDRS III as well as bradykinesia sub-scores in response to acute levodopa challenge (Figures 4C, D). Following levodopa administration, no correlations between gaze range and MDS-UPDRS III scores or sub-scores were identified among either PSP or MSA subjects.

## 4 Discussion

Here, we noted a reduced maximum range of ocular motion for both vertical and horizontal gaze in people among PD patients. This deficit could be corrected by acute levodopa challenge. Oculomotor range negatively correlated with MDS-UPDRS III scores; furthermore, the ratio of improvement in maximal oculomotor range was noted to correlate with improvement of MDS-UPDRS III scores and bradykinesia sub-scores after levodopa treatment. Although the maximum range of ocular movement was also impaired in PSP and MSA patients, PSP patients exhibited the most pronounced impairment relative to PD and MSA patients. Reduced range of upward gaze was determined to be the most sensitive indicator for distinguishing between the aforementioned three parkinsonian syndromes, gaze was most prominently impaired among PSP patients, the best corrective effect of levodopa was noted among in PD patients, and an almost normal maximal range of ocular motion noted among MSA patients. To the best of our knowledge, this is the first research that quantitatively assessed the impaired maximum range of ocular movement and explored the impact of levodopa on ocular movements in individuals with PD. Reduced maximum oculomotor range, especially along the vertical axis, has long been considered as one of the clinical hallmarks of PSP (Leigh et al., 2010). However, whether the maximum range of eye movement was altered in PD has not been previously studied. Here, we found that the maximum oculomotor range decreased for both vertical and horizontal gaze in PD patients. Importantly, the range of maximum gaze was found to negatively correlate with MDS-UPDRS III scores and improved after levodopa administration in a subgroup of PD patients, suggesting that this alteration in ocular range was associated to the underlying pathological degeneration of the dopaminergic system. Moreover, improvement in maximal downward gaze was found to associate with improvement in MDS-UPDRS III scores and bradykinesia sub-scores, further supporting our findings.

Although prior studies detailed hypometric saccades, reduced saccade velocity, and prolonged saccade latency among PD patients (Zhou et al., 2022; Li et al., 2023), our findings further confirm the pathophysiological similarity of limb bradykinesia to distinctive manifestations clinically noted in ocular muscles. However, ocular muscle rigidity or the influence of other yet-unidentified pathophysiological mechanisms cannot be excluded from influencing ocular movement in the setting of PD.

Although we noted abnormalities in ocular movement in PSP and MSA patients, they were nevertheless distinct from those noted in PD patients. While PSP patients exhibited more severe deficits relative to PD and MSA patients, no significant difference was detected between MSA and PD patients despite a trend of more pronounced impairment among MSA patients relative to those suffering PD. This finding is in agreement with prior research, suggesting that abnormal voluntary eye movements are often more severe in PSP and relatively less impacted in PD and MSA (Valls-Solé et al., 1997; Armstrong, 2021). Importantly, maximal ranges of ocular motion on downward and horizontal gazes were impaired in all three patient groups in relation to the HC group, indicating that moderate limitations in oculomotor range should be cautiously considered when establishing a differential diagnosis. In contrast to PD patients, levodopa only partially improved deficits in maximal oculomotor range among PSP and MSA patients. It is of note that, the maximal angle of upward gaze was most prominently impaired in PSP patients and less so in PD, in contrast to MSA patients. Furthermore, levodopa challenge only corrected upward gaze in PD but not PSP patients. The range of vertical ocular movement and its response to levodopa challenge may serve as a more sensitive indicator for differentiating parkinsonian disorders. Marked reduction of range in upward gaze but poor response to levodopa thus supports the diagnosis of PSP. Indeed, upward gaze palsy was reported to be more frequent than downward gaze palsy in PSP-RS patients (Leigh et al., 2010). The relatively lower efficacy of levodopa in correcting the impaired oculomotor range in PSP and MSA patients suggests that the underlying mechanism of oculomotor deficits in atypical parkinsonian disorders is distinct from that in classical PD. Notably, vertical gaze limitation in the setting of PSP was reported to be caused by supranuclear palsy, which likely associates with neurodegeneration in the tectum of the midbrain (Quattrone et al., 2016; Buch et al., 2022).

Previous research has reported a decline in the range of gaze elevation as age advances (Chamberlain, 1971; Clark and Isenberg, 2001). In the current investigation, we observed negative associations between age and eye movement ranges in all groups, thus corroborating prior findings. The mean age of PSP patients in our study was greater as compared to that of other groups; this disparity may have influenced our findings. However, other studies have reported that downward gaze is unaffected by increasing age (Lee et al., 2019); hence, the reduced maximum downward angle in PSP patients found in this study may also accurately reflect oculomotor function. Indeed, the patients included in our PSP group comprised both PSP-RS and PSP-P patients. Previously, PSP-P patients were reported to exhibit greater preservation of vertical saccadic function as compared to PSP-RS patients on quantitative evaluation (Pagonabarraga et al., 2021). Although ocular movement deficits in PSP-RS patients have been extensively characterized, there remains a lack of comprehensive data for PSP-P and other disease subtypes. The noted disparities in eye movement patterns among different PSP subtypes warrant consideration of treating each subtype as an independent patient group in future studies.

The oculomotor angles have displayed variability in previous measurements mainly ascribed to differences in methodology, measuring instruments or other confounding factors. Generally, the elevation angle ranged from 25–40°, depression from 40–60°, adduction from 40–60°, and abduction from 40–60° (Chamberlain, 1971; Gerling et al., 1997; Lim et al., 2014). The outcomes presented within this study exhibit numerically higher values when compared with earlier research due to methodological advancements employed here along with the use of binocular measurements rather than monocular ones. Here, the maximum range of ocular movement was measured using a specially-developed method. Instrumentation was modified from kinetic perimetry testing equipment and used in peripheral visual field testing. The main differences between this study's method and conventional kinetic perimetry are as follows: (1) a cross-shaped marked scale consisting of vertical and horizontal sections affixed to the wall replaces a bowl or arch-shaped visual target; (2) the subject's head is kept steady, but eye movements in evaluated directions are encouraged to be made with maximal effort rather than maintaining sustained fixation of gaze on the zero point during testing; (3) ocular movements of both eyes are checked simultaneously instead of separately; and (4) oculomotor range is evaluated only in four directions (i.e., upward and downward vertically and leftward and rightward horizontally). The maximal oculomotor distance in this study was considered as that of both eyes as a unit. We approximated the actual length between the eyes and the zero point as a fixed length of 30 cm (slightly shorter than the distance from each eye to the zero point on the marked scale), which could lead to conversion angle overestimation. Such slight systematic bias, however, was considered predictable and to not affect relative differences between various subjects; importantly, this adaptation greatly facilitated testing procedure. In summary, methodological modification was found to improve testing device usability and efficiency, making it suitable for daily clinical practice. Prior to experimentation, test-retest reliability was assessed in a cohort of 30 healthy individuals at an interval of 1 week by the same evaluator. The test-retest reliability was deemed satisfactory since the correlation coefficients were above 0.50 for maximal oculomotor angles in all directions.

Several limitations exist in this study. This study was not without limitations. Firstly, the relatively small number of subjects with PSP and MSA posed challenges in conducting a comprehensive subgroup analysis for different disease phenotypes; our findings thus warrant further study considering larger patient samples. Secondly, as the diagnoses of various parkinsonian subtypes were made based on clinical features and without pathological confirmation, potential misdiagnoses could not be excluded. Long-term follow-up is necessary to further validate clinical diagnosis among certain patients. Finally, as only relevant parameters were retested 1 hour after levodopa challenge, the effects of levodopa treatment may have been less than optimally reflected. Further studies should aim to extend the duration of levodopa challenge in both classical and atypical PD patient groups.

## 5 Conclusion

In conclusion, we found a reduction in both vertical and horizontal maximum range of ocular motion among PD patients. These deficits were ameliorated by levodopa treatment. The maximum range of upward gaze and the beneficial effects of levodopa may assist clinical differential diagnosis of parkinsonian subtypes. The most prominent impairment was noted in PSP patients and the best response to levodopa challenge was noted in PD patients; an almost normal vertical gaze range of motion was noted in MSA-P patients. A moderate reduction of oculomotor range should be considered with caution when differentiating between various parkinsonian subtypes.

## Data availability statement

All raw data of this study concerning the participants may be made available upon request to the corresponding author.

## Ethics statement

The studies involving humans were approved by the Ethical Committee of the First Affiliated Hospital of Anhui Medical University. The studies were conducted in accordance with the local legislation and institutional requirements. The participants provided their written informed consent to participate in this study.

## Author contributions

JL: Conceptualization, Formal analysis, Writing—original draft. YL: Data curation, Investigation, Methodology, Writing—review & editing. XChu: Data curation, Supervision, Writing—original draft. MJ: Methodology, Validation, Writing—original draft. TW: Resources, Supervision, Writing—review & editing. XChe: Conceptualization, Funding acquisition, Validation, Writing—review & editing.
